# Alterations in brain structure associated with trigeminal nerve anatomy in episodic migraine

**DOI:** 10.3389/fpain.2022.951581

**Published:** 2022-07-18

**Authors:** Tiffani J. Mungoven, Noemi Meylakh, Vaughan G. Macefield, Paul M. Macey, Luke A. Henderson

**Affiliations:** ^1^School of Medical Sciences (Neuroscience), Brain and Mind Centre, University of Sydney, Camperdown, NSW, Australia; ^2^Department of Anatomy and Physiology, University of Melbourne, Melbourne, VIC, Australia; ^3^UCLA School of Nursing and Brain Research Institute, University of California, Los Angeles, CA, United States

**Keywords:** diffusion tensor imaging, MRI, PAG, brain imaging, mean diffusivity, fractional anisotropy

## Abstract

The pathophysiology of migraine remains to be elucidated. We have recently shown that interictal migraineurs exhibit reduced fractional anisotropy (FA) in the root entry zone of the trigeminal nerve when compared to controls, but it is not known if this altered nerve anatomy is associated with changes within the brainstem or higher cortical brain regions. Diffusion tensor imaging of the brain was used to calculate regional measures of structure, including mean diffusivity (MD), axial diffusivity (AX) and radial diffusivity (RD) in addition to voxel-based morphometry of T1-weighted anatomical images. Linear relationships between trigeminal nerve anatomy (FA) and MD throughout the brainstem and/or higher cortical regions were determined in both controls (*n* = 31, brainstem; *n* = 38, wholebrain) and interictal migraineurs (*n* = 32, brainstem; *n* = 38, wholebrain). Additionally, within the same brain areas, relationships of AX and RD with nerve FA were determined. We found that in both interictal migraine and control participants, decreasing trigeminal nerve FA was associated with significantly increased MD in brainstem regions including the spinal trigeminal nucleus and midbrain periaqueductal gray matter (PAG), and in higher brain regions such as the hypothalamus, insula, posterior cingulate, primary somatosensory and primary visual (V1) cortices. Whereas, both control and migraineur groups individually displayed significant inverse correlations between nerve FA and MD, in migraineurs this pattern was disrupted in the areas of the PAG and V1, with only the control group displaying a significant linear relationship (PAG controls *r* = –0.58, *p* = 0.003; migraineurs *r* = –0.25, *p* = 0.17 and V1 controls *r* = −0.52, *p* = 0.002; migraineurs *r* = –0.10, *p* = 0.55). Contrastingly, we found no gray matter volume changes in brainstem or wholebrain areas. These data show that overall, trigeminal nerve anatomy is significantly related to regional brain structure in both controls and migraineurs. Importantly, the PAG showed a disruption of this relationship in migraineurs suggesting that the anatomy and possibly the function of the PAG is uniquely altered in episodic migraine, which may contribute to altered orofacial pain processing in migraine.

## Introduction

Migraine is a recurrent neurological disorder, characterized by unilateral headaches of moderate to severe pain intensity that are typically accompanied by symptoms of nausea, vomiting, photophobia and phonophobia (1). Although our understanding about migraine pathophysiology has evolved over the last several decades, the precise underlying neural mechanisms remain to be uncovered. While an ongoing debate has ensued about whether migraine attacks originate from peripheral triggers vs. central brain changes, it is likely that a synergistic relationship between peripheral and central mechanisms is critical for migraine generation (2).

Given that the pain associated with migraine is primarily located within the trigeminal nerve distribution, it is likely that there may be disturbances in processing within the spinal trigeminal nucleus (SpV), the site at which orofacial noxious afferents first terminate. We have recently reported altered trigeminal nerve anatomy in individuals with episodic migraine, characterized by significantly reduced fractional anisotropy (FA) in the root entry zone (3). This FA reduction is consistent with histological findings of structural abnormalities in the myelin sheath and axons of the zygomaticotemporal branch of the trigeminal nerve in episodic migraine (4). Why and how these peripheral nerve structural changes occur remains unknown, but these alterations suggest that peripheral processes contribute to migraines. It is likely that anatomical changes in the trigeminal nerve result in changes within the brainstem where the trigeminal nerve terminates and potentially other higher processing regions involved in orofacial sensory processing. Indeed, we have previously reported anatomical changes in the brainstem in episodic migraine including increased mean diffusivity (MD) in the SpV, dorsolateral pons (dlPons) and midbrain periaqueductal gray matter (PAG) (5).

How such changes within the brain occur in migraine is unknown. It has been hypothesized that the internal state of the migraineur's brain creates a high allostatic load leading to progressively damaging brain function and structure, which worsens as the frequency of migraines increases, plunging the individual into what has been coined, “*a feedforward cascade”* (6). In addition, it may be the case that some of the central anatomical changes are associated with changes in the anatomy of the trigeminal nerve. Such a relationship between peripheral and central neural anatomy may be present in controls and may be altered in migraineurs as the nerve structure becomes altered. Indeed, this relationship may represent subtle adaptive changes that occur at the cellular level within the nerve and associated brain areas during orofacial processing under normal circumstances (6).

The aim of this study is to extend our previous investigation (3) to determine whether changes in FA of the trigeminal nerve are associated with microstructural alterations in the brainstem and/or higher cortical brain regions in migraine participants, measured using diffusion weighted imaging and voxel-based morphometry of T1-weighted anatomical images. We hypothesize that increasing degrees of microstructural changes in the trigeminal nerve (measured as FA) will be associated with greater structural changes (measured as MD) and alterations in gray matter volume in brainstem orofacial pain processing regions such as the SpV and PAG. In addition, we hypothesize that these anatomical changes will also be transferred to higher pain processing regions such as the hypothalamus and primary somatosensory cortex. This transference may occur through persistent activation and peripheral sensitization of nociceptors leading to adaptive changes in secondary afferents which propagate this central sensitization to higher cortical areas involved in pain perception (7, 8).

## Materials and methods

### Participants

Thirty-eight participants with migraine (29 females; mean ± SEM age, 30.2 ± 1.5 years) and 38 pain-free controls (30 females; mean ± SEM age, 31.0 ± 1.6 years) were recruited for the study from the general population using an advertisement. There was no significant difference in age (*t-*test; *p* > 0.05) or gender distribution (χ^2^ test, *p* > 0.05) between the two groups. Episodic migraine participants were diagnosed according to the criteria laid out by the International Classification of Headache Disorders (ICHD), 3rd edition, sections 1.1 and 1.2 (1). Nine migraine participants reported experiencing aura associated with their migraines and the remaining 29 reported no aura. All migraine participants underwent MRI scanning during a pain-free interictal period, that is, not within the 72 h after or the 24 h prior to a migraine event. Data from all the participants used in this investigation were also included in our previous study (3) and more than half of the migraine participants were also used in our previous studies (5, 9–12).

The criteria for the exclusion of control participants in the study were the presence of any current pain or chronic pain condition, current use of analgesics or any neurological disorder. The exclusion criteria for migraine participants were any other pain condition other than migraine or any other neurological disorder. No migraineur was excluded based on their medication use and no migraine or control participant had an incidental neurological finding during routine MRI screening by the radiologist. All migraine participants indicated the migraine pain intensity (6-point visual analog scale; 0 = no pain, 5 = most intense imaginable pain) and drew the facial distribution of pain they commonly experienced during a migraine attack. In addition, each participant described the qualities of their migraines and indicated any current treatments used to prevent or abort a migraine once initiated. Informed written consent was obtained for all procedures according to the Declaration of Helsinki seventh revision and local Institutional Human Research Ethics Committee approved the study.

### MRI acquisition

Participants lay supine on the bed of a 3T MRI scanner (Phillips, Achieva) with their head immobilized in a close-fitting 32-channel head coil; padding was used to limit head movement. In each of the 38 migraine and 38 control participants, a high-resolution 3D T1-weighted anatomical image set covering the entire brain was collected (turbo field echo; echo time = 2.5 ms, repetition time = 5,600 ms, flip angle = 8°, voxel size = 0.87 × 0.87 × 0.87 mm). Following this, a high-resolution diffusion tensor image (DTI) set covering the entire brain was collected using a single-shot, multi section, spin-echo echo-planar pulse sequence (repetition time = 8,788 ms; flip angle = 90°, matrix size = 112 × 112, field of view = 224 × 224 mm, slice thickness = 2.5 mm, 55 axial slices). For each slice, diffusion gradients were applied along 32 independent orientations with *b* = 1,000 s/mm^2^ after the acquisition of *b* = 0 s/mm^2^ (b0) images. Anatomical and DTI image sets were visually inspected for artifacts (ghosting, distortion, blurring and/or signal dropout); no subject was excluded.

### Image processing

#### DTI analysis

Using SPM12 (13) and custom Matlab software, corrections for eddy current and b0 distortions were performed, the DTI image sets from each participant were realigned based on the b0 images and the diffusion tensors were calculated at each voxel from the images using a linear model. The diffusion tensor was calculated using the method proposed by Basser and Pierpaoli (14). Once the elements of diffusion tensor were calculated, mean diffusion (MD), axial diffusion (AX) and radial diffusion (RD) wholebrain maps were derived and co-registered to each individual participant's T1-weighted anatomical image. The T1 images were then spatially normalized to the Montreal Neurological Institute (MNI) template and the normalization parameters were applied to the DTI images to place them in MNI space. The wholebrain images were smoothed using a 5 mm full-width at half maximum (FWHM) Gaussian filter.

Additionally, prior to spatial normalization, using brainstem-specific isolation software (SUIT toolbox) (15), a mask of the brainstem was manually created on each of the participant's T1-weighted anatomical image set and each of the participant's DTI image set (Supplementary Figure 1). Using these masks, the brainstem was isolated from the MD, AX and RD maps, spatially normalized and re-sliced to the SUIT brainstem template in MNI space. These brainstem-only image sets were spatially smoothed using a 3 mm FWHM Gaussian filter. Seven control and 6 migraine participants were excluded from the brainstem-only analysis due to their DTI images not extending over the caudal brainstem. In all analyses, the anatomical location of each significant cluster was identified using the Atlases of the Human Brain (16) and the Human Brainstem (17).

### Brainstem specific FA left trigeminal nerve covariate analysis

**Figure 1:**
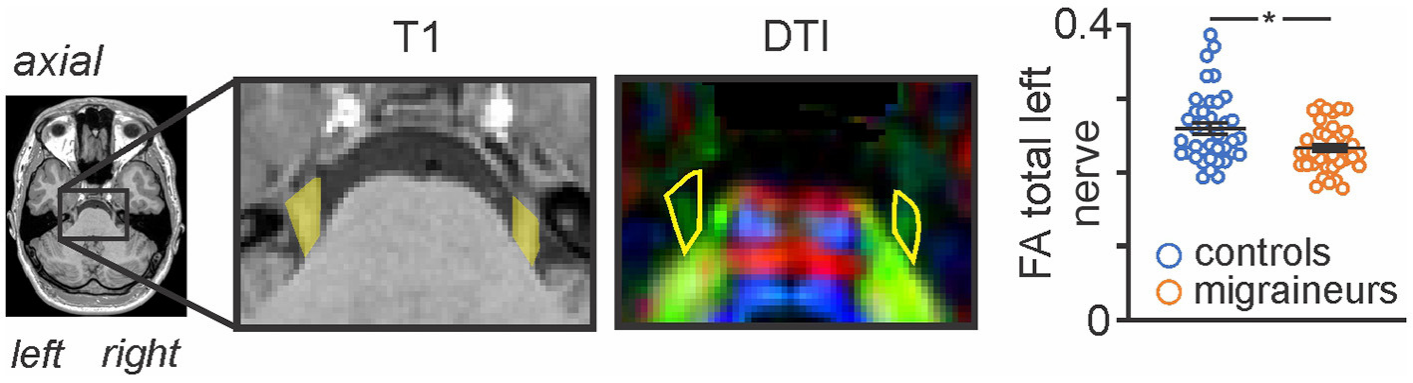
Axial T1-weighted anatomical image and corresponding diffusion tensor (DTI) image showing the trigeminal nerve root entry zone in a single participant. The DTI image is color-coded for direction of greatest water movement. The outline of the trigeminal nerve region used for the total nerve analysis is shown in yellow shading on the T1-weighted anatomical image and is also outlined in yellow on the DTI images. This volume of interest encompassing the root entry zone is the section of the trigeminal nerve that lies within the pontine cistern, i.e., from where it emerges from the pons, to the point at which it exits the pontine cistern anteriorly. Plots of FA left nerve values for individual controls and migraineurs are shown in the right inset. Horizontal bars indicate mean (±SEM) in the control and migraine groups.

Given that we have previously found the left trigeminal nerve root entry zone of the migraine group to be significantly lower than the left nerve of the control group for FA only (3) (Figure 1), we chose to extend our previous investigation to assess whether there were covariations between FA of the left trigeminal nerve and any regional brainstem diffusion parameters (MD, AX, RD). A second level, voxel-by-voxel analysis with nerve FA as a covariate of interest was performed with the brainstem specific MD images on all controls (*n* = 31) and interictal migraineurs (*n* = 32) (*p* < 0.05, false discovery rate corrected (FDR) at a single voxel level, minimum cluster size 5 contiguous voxels, age and gender nuisance variables, a brainstem mask that excluded cerebrospinal fluid as well as the cerebellum was applied to each analysis). Significant clusters were overlaid onto a standard brainstem template for visualization.

To assess the influence of group on linear relationships we extracted MD values for each significant cluster and plotted them against nerve FA for the control and migraine groups separately and assessed significance (Fisher r-to-z transformation, *p* < 0.05). In addition, to assess if there were overall MD differences between the control and migraine groups, MD values for each of the significant clusters were extracted, the mean ± SEM plotted, and significance determined using two-tailed, two-sample *t-*tests (*p* < 0.05). In addition, AX and RD values were extracted from each of the significant clusters and plotted and significant differences between control and migraine groups determined (two-tailed, two-sample *t-*test, *p* < 0.05, corrected for multiple comparisons for 3 regions of interest).

### Wholebrain FA left trigeminal nerve covariate analysis

Using the wholebrain images, we again assessed the relationship between FA of the left trigeminal nerve and the diffusion parameters (MD, AX, RD) in the same analysis procedures described above for controls (*n* = 38) and migraineurs (*n* = 38) (*p* < 0.05, FDR, minimum cluster size 10 contiguous voxels and age and gender were included as nuisance variables). Linear relationships between each of these significant clusters and left trigeminal nerve FA values for the total control and migraine groups were determined. Significant clusters were overlaid onto a mean T1 anatomical image set. MD values were extracted from significant clusters, plotted against trigeminal nerve FA for the control and migraine groups separately and significance assessed (Fisher r-to-z transformation, *p* < 0.05). In addition, overall MD differences between the control and migraine groups were plotted and significance determined (two-tailed, two-sample *t-*test, *p* < 0.05). Finally, AX and RD values were extracted, plotted and significant group differences determined (two-tailed, two-sample *t-*test, *p* < 0.05, corrected for multiple comparisons for 6 regions of interest).

#### Voxel based morphometry analysis

Using SPM12, T1-weighted anatomical image sets were segmented and spatially normalized, resulting in brainstem and wholebrain “maps” of gray matter probabilities spatially normalized into MNI space and modulated by the volume changes due to the normalization (probability × volume), i.e., maps of regional gray matter volume. The normalized, modulated gray matter maps were smoothed using a 3 mm and 5 mm FWHM for the brainstem and wholebrain, respectively. We assessed the relationship between FA of the left trigeminal nerve and gray matter volumes using the same analysis procedures described above for controls (*n* = 38) and migraineurs (*n* = 38) (*p* < 0.05, FDR, minimum cluster size wholebrain 10 contiguous voxels, brainstem 5 contiguous voxels, age and gender were included as nuisance variables). Since we found no significant clusters in either the brainstem or wholebrain analysis, we also extracted gray matter volumes from each of the significant derived clusters from the wholebrain and brainstem mean diffusivity analysis described above. For each cluster, gray matter volumes were extracted and plotted against trigeminal nerve FA for the control and migraine groups separately and significance assessed for both the brainstem and wholebrain (Fisher r-to-z transformation, *p* < 0.05). In addition, overall gray matter volume differences between the control and migraine groups were plotted and significance determined (two-tailed, two-sample *t-*test, *p* < 0.05).

## Results

### Migraine characteristics

Using a self-report questionnaire, migraine participants most frequently described their migraine pain as “throbbing,” “pulsating,” and/or “sharp” in nature. They indicated that “stress,” “lack of sleep,” and/or “bright light” most often triggered their migraine attacks. The mean (±SEM) length of time since the onset of migraine attacks (years suffering) was 15.9 ± 2.0 years, the mean estimated frequency of migraine attacks was 1.9 ± 0.4 per month, and the mean pain intensity of migraines was 3.8 ± 0.2, measured by a 6-point visual analog scale. Although 23 of 38 were taking some form of daily medication (mostly the oral contraceptive pill; 14 migraineurs), none of the migraine participants were taking prophylactic medication for migraine.

#### Diffusion value changes

##### Brainstem specific FA left trigeminal nerve covariate analysis

**Figure 2:**
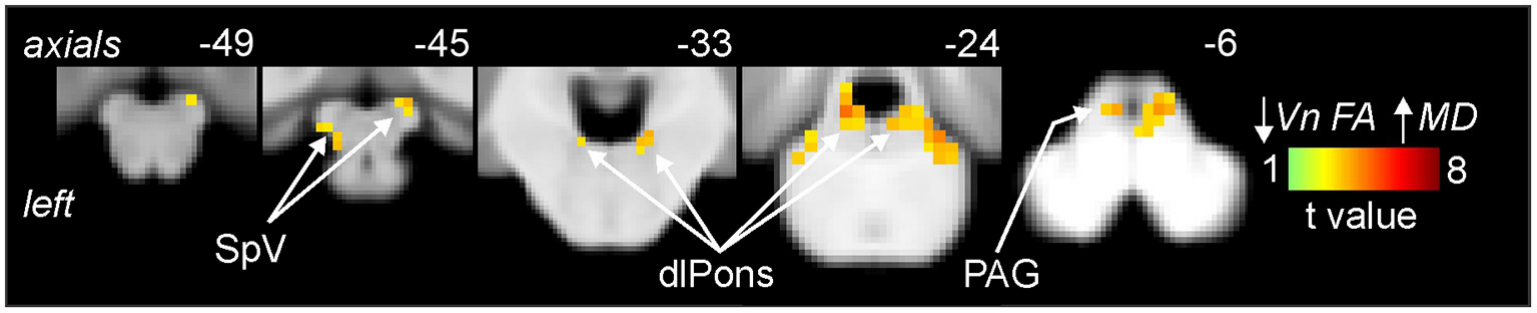
Areas of the brainstem in which reductions in trigeminal nerve fractional anisotropy (FA) were significantly correlated with increases in brainstem mean diffusivity (MD) assessed in migraine (*n* = 32) and control participants (*n* = 31). Significant clusters are overlaid onto a mean T1-weighted brainstem template image. Slice locations in Montreal Neurological Institute (MNI) space are indicated to the top right of each slice. Note that MD was significantly correlated to nerve FA in the brainstem regions of the spinal trigeminal nucleus (SpV), dorsolateral pons (dlPons) and the periaqueductal gray matter (PAG).

As previously reported, we found that migraine was associated with a significant decrease in FA of the left trigeminal nerve in the same participants used in this study [see (3) for analysis details]. Figure 1 shows the area of the trigeminal nerve sampled and also a plot of the mean (±SEM) and individual FA values for the trigeminal nerve in all 38 control and 38 migraine participants (mean ± SEM FA: controls 0.26 ± 0.01; migraineurs 0.23 ± 0.01, *p* = 0.01 two-tailed, two-sample *t-*test) (see Supplementary Figure 1 for more trigeminal nerve samples). Brainstem analysis revealed that decreasing trigeminal nerve FA was associated with increases in MD in a number of brainstem regions bilaterally, namely the spinal trigeminal nucleus (SpV), dorsolateral pons (dlPons) and the midbrain periaqueductal gray matter (PAG) (Figure 2; Table 1). That is, the lower the trigeminal nerve FA (indicative of damage), the greater the MD in brainstem sites.

**Table 1:** Montreal Neurological Institute (MNI) coordinates, cluster size and *t-*score for regions in which decreasing trigeminal nerve fractional anisotropy was associated with increases in mean diffusivity in control and interictal migraine participants.

**Brain region**	**MNI Co-ordinate**			**cluster size**	***t*** **-score**
	**x**	**y**	**z**		
**Brainstem**					
Left spinal trigeminal nucleus	−6	−42	−39	30	4.16
Right spinal trigeminal nucleus	10	−30	−43	24	3.96
Right/left dorsolateral pons	6	−38	−19	186	5.33
Right dorsolateral pons	4	−42	−39	11	3.74
Right/left periaqueductal gray matter	−4	−32	−3	26	4.33
**Wholebrain**					
Left posterior cingulate cortex	−8	−30	38	1,490	5.71
Left primary somatosensory cortex	−44	−10	26	37	3.67
Left dorsoposterior insula	−36	−8	10	69	3.55
Right/left hypothalamus	2	−12	−10	24	3.68
Left putamen	−28	−2	2	33	3.91
Right/left primary visual cortex	0	−81	12	56	4.27

**Figure 3:**
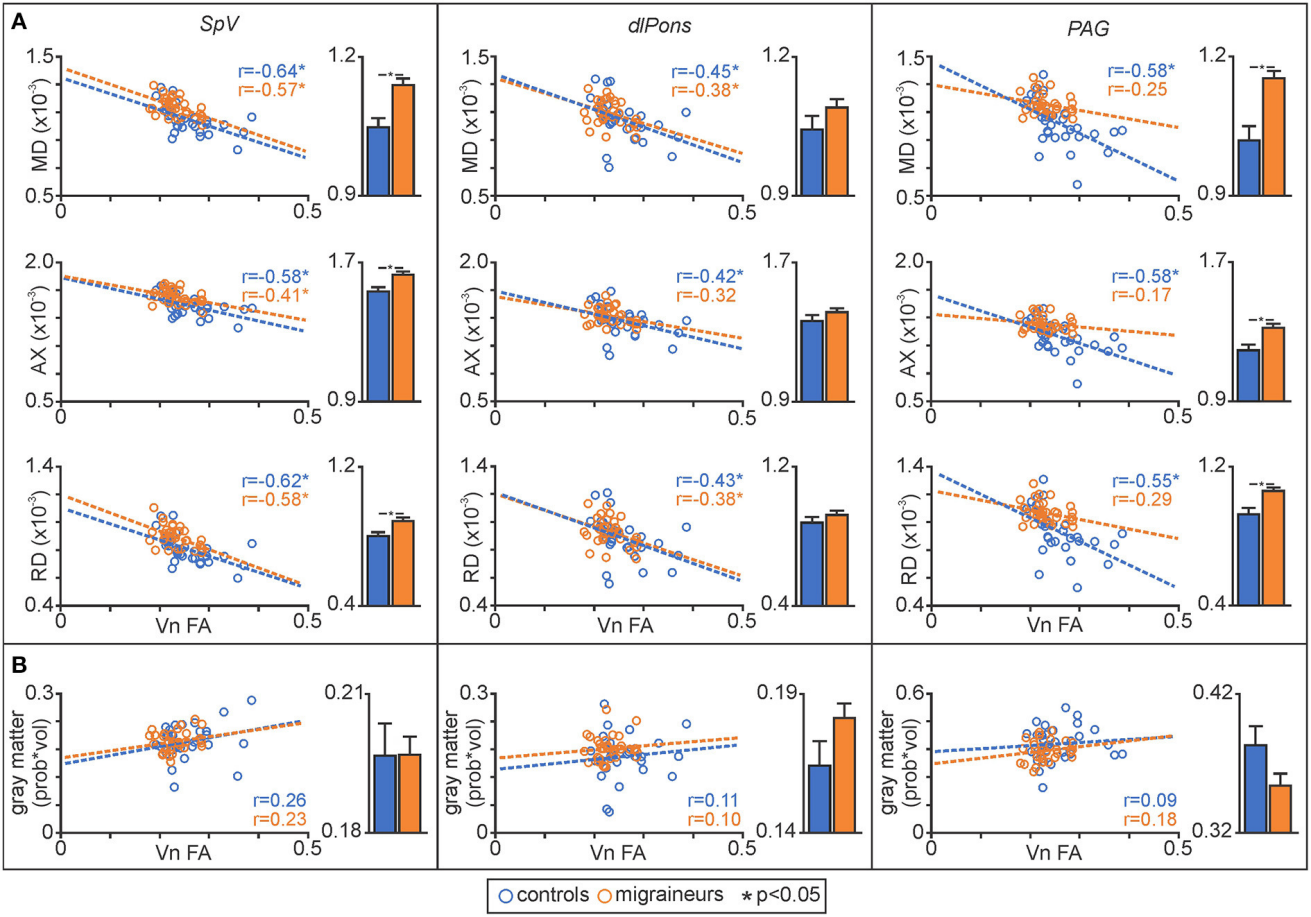
**(A)** Plots of mean diffusivity (MD), axial diffusivity (AX) and radial diffusivity (RD) against trigeminal nerve fractional anisotropy (FA) for the spinal trigeminal nucleus (SpV), dorsolateral pons (dlPons) and the periaqueductal gray matter (PAG). In addition, to the right of each correlation plot are plots of mean (±SEM) MD, AX and RD values for each of the significant brainstem clusters. **(B)** Plots of gray matter volume against trigeminal nerve FA derived from significant MD clusters and plots of mean (±SEM) gray matter volume for each of the significant MD brainstem clusters.

Plots of trigeminal nerve FA values against MD revealed that both control and migraineur groups displayed significant negative correlations in the SpV (controls *r* = –0.64, *p* = 0.001, migraineurs *r* = –0.57, *p* = 0.002) and dlPons (controls *r* = –0.45, *p* = 0.02; migraineurs *r* = –0.38, *p* = 0.04). In contrast, in the PAG only the controls displayed a significant relationship whereas the migraineurs did not (controls *r* = –0.58, *p* = 0.003; migraineurs *r* = –0.25, *p* = 0.17) (Figure 3). Similar relationships were observed between nerve FA with AX and RD for all three brainstem regions.

In addition, to assessing relationships between nerve FA and brainstem MD, for each significant cluster we compared MD, AX and RD values between controls and migraineurs and found that overall, migraineurs had higher values than controls in the SpV and the PAG (Figure 3; Table 2). In contrast, there was no significant difference between controls and migraineurs for any of the diffusivity measures for the dlPons.

**Table 2:** Diffusion and *p-*values for significant brainstem clusters in migraineurs and controls.

	***SpV***	***dlPons***	***PAG***
**Mean diffusivity [mean (±SEM) x10** ^**−3**^ **]**			
Controls	1.05 ± 0.02	1.04 ± 0.03	1.02 ± 0.03
Migraineurs	1.14 ± 0.01	1.09 ± 0.02	1.15 ± 0.01
Significance	*p =* 0.0001	*p =* 0.13	*p <* 0.0001
**Axial diffusivity [mean (±SEM) x10** ^**−3**^ **]**			
Controls	1.54 ± 0.02	1.37 ± 0.03	1.20 ± 0.03
Migraineurs	1.63 ± 0.01	1.42 ± 0.02	1.33 ± 0.01
Significance	*p =* 0.0002	*p =* 0.11	*p =* 0.0001
**Radial diffusivity [mean (±SEM) x10** ^**−3**^ **]**			
Controls	0.81 ± 0.02	0.88 ± 0.03	0.93 ± 0.03
Migraineurs	0.89 ± 0.01	0.93 ± 0.02	1.07 ± 0.01
Significance	*p =* 0.0003	*p =* 0.17	*p =* 0.0001

*Note that in the SpV and PAG, migraineurs displayed significantly greater mean diffusivity values compared to controls for all three diffusivity measures (corrected for multiple comparisons)*.

##### Wholebrain FA left trigeminal nerve covariate analysis

**Figure 4:**
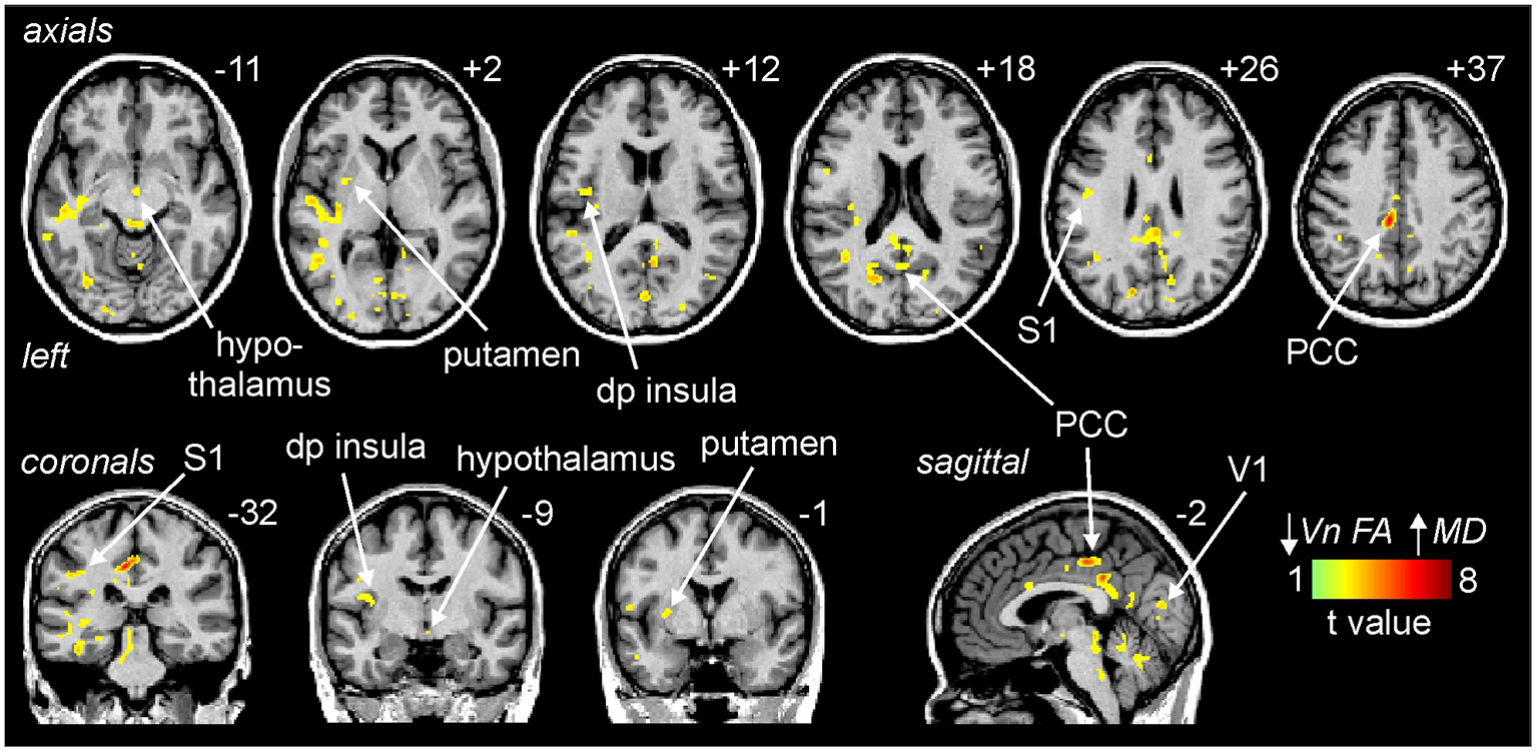
Areas above the brainstem in which reductions in trigeminal nerve fractional anisotropy (FA) were significantly correlated with increases in above the brainstem mean diffusivity (MD) assessed in migraine (*n* = 38) and control participants (*n* = 38). Significant clusters are overlaid onto a mean T1-weighted anatomical image. Slice locations in Montreal Neurological Institute (MNI) space are indicated to the top right of each slice. Note that MD was significantly correlated to nerve FA in the posterior cingulate cortex (PCC), primary somatosensory cortex (S1), dorsoposterior insula (dp insula), hypothalamus, putamen and the primary visual cortex (V1).

Similar to the brainstem, decreasing trigeminal nerve FA was associated with increases in MD in a number of sites above the level of the brainstem. These significant inverse linear correlations were present in the posterior cingulate cortex (PCC), primary somatosensory cortex (S1), dorsoposterior insula (dp insula), hypothalamus, putamen and primary visual cortex (V1) (Figure 4; Table 1).

**Figure 5:**
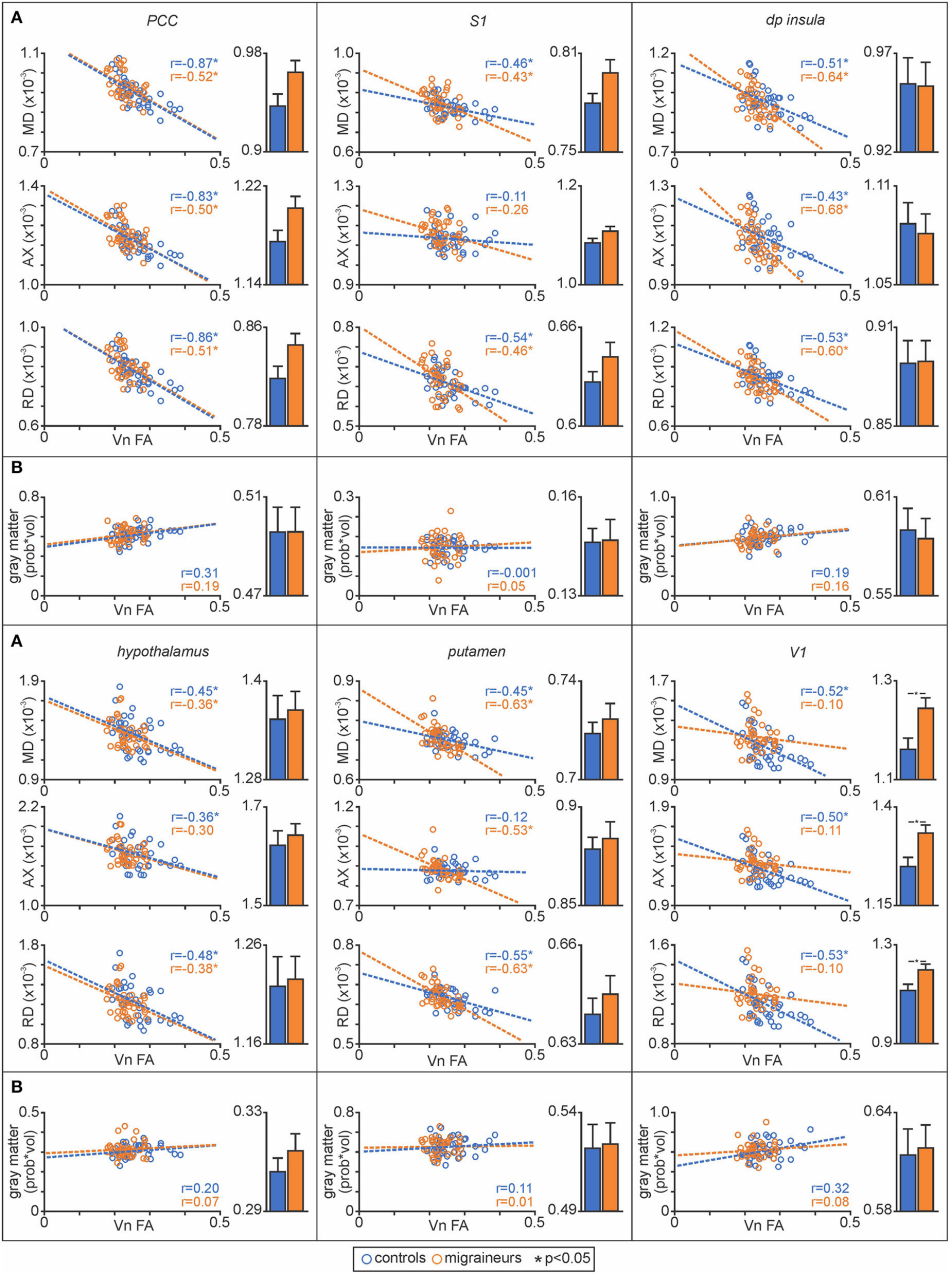
**(A)** Plots of mean diffusivity (MD), axial diffusivity (AX) and radial diffusivity (RD) against trigeminal nerve fractional anisotropy (FA) for the posterior cingulate cortex (PCC), primary somatosensory cortex (S1), dorsoposterior insula (dp insula), hypothalamus, putamen and primary visual cortex (V1). In addition, to the right of each correlation plot are plots of mean (±SEM) MD, AX and RD values for each of the significant above the brainstem clusters. **(B)** Plots of gray matter volume against trigeminal nerve FA derived from significant MD clusters and plots of mean (±SEM) gray matter volume for each of the significant MD above the brainstem clusters.

Plots of nerve FA against MD revealed that both control and migraineur groups displayed significant negative correlations in the PCC (controls *r* = –0.87, *p* < 0.0001, migraineurs *r* = –0.52, *p* = 0.003), S1 (controls *r* = –0.46, *p* = 0.01; migraineurs *r* = –0.43, *p* = 0.01), dp insula (controls *r* = –0.51, *p* = 0.003; migraineurs *r* = –0.64, *p* = 0.0002), hypothalamus (controls *r* = –0.45, *p* = 0.01; migraineurs *r* = –0.36, *p* = 0.03) and putamen (controls *r* = –0.45, *p* = 0.01; migraineurs *r* = –0.63, *p* = 0.0003) (Figure 5). In V1, only the controls displayed a significant correlation (controls *r* = –0.52, *p* = 0.002; migraineurs *r* = –0.10, *p* = 0.55). Similar relationships were observed between nerve FA with AX and RD for all of these wholebrain regions.

Extraction of MD values from these significant clusters revealed no overall group differences in MD, AX or RD between controls and migraineurs in the PCC, S1, dp insula, hypothalamus and putamen (Figure 5; Table 3). In contrast, all three diffusivity measures were significantly greater in migraineurs compared with controls in V1.

**Table 3:** Diffusion and *p-*values for significant wholebrain clusters in migraineurs and controls.

	***PCC***	***S1***	***dp insula***	***hypothalamus***	***putamen***	***V1***
**Mean diffusivity [mean (±SEM) x10^−3^]**						
controls	0.94 ± 0.01	0.78 ± 0.01	0.95 ± 0.01	1.35 ± 0.03	0.72 ± 0.004	1.16 ± 0.02
migraineurs	0.96 ± 0.01	0.80 ± 0.01	0.95 ± 0.01	1.37 ± 0.02	0.72 ± 0.01	1.25 ± 0.02
significance	*p =* 0.04	*p =* 0.05	*p =* 0.95	*p =* 0.75	*p =* 0.42	*p =* 0.005
**Axial diffusivity [mean (±SEM) x10^−3^]**						
controls	1.18 ± 0.01	1.09 ± 0.01	1.09 ± 0.01	1.62 ± 0.03	0.88 ± 0.01	1.25 ± 0.02
migraineurs	1.20 ± 0.01	1.11 ± 0.01	1.08 ± 0.01	1.64 ± 0.02	0.88 ± 0.01	1.33 ± 0.02
significance	*p =* 0.03	*p =* 0.04	*p =* 0.72	*p =* 0.57	*p =* 0.59	*p =* 0.005
**Radial diffusivity [mean (±SEM) x10^−3^]**						
controls	0.82 ± 0.01	0.63 ± 0.01	0.89 ± 0.01	1.22 ± 0.03	0.64 ± 0.005	1.12 ± 0.02
migraineurs	0.85 ± 0.01	0.64 ± 0.01	0.89 ± 0.01	1.23 ± 0.02	0.65 ± 0.01	1.20 ± 0.02
significance	*p =* 0.04	*p =* 0.14	*p =* 0.95	*p =* 0.85	*p =* 0.39	*p =* 0.006

*Note that only the V1 was significantly greater in migraineurs compared to controls for all three diffusivity measures (corrected for multiple comparisons)*.

#### Gray matter volume changes

In contrast, decreasing trigeminal nerve FA was not associated with any changes in gray matter volume in brainstem or wholebrain regions; SpV (controls *r* = 0.26, *p* = 0.16, migraineurs *r* = 0.23, *p* = 0.22), dlPons (controls *r* = 0.11, *p* = 0.55; migraineurs *r* = 0.10, *p* = 0.59), PAG (controls *r* = 0.09, *p* = 0.63; migraineurs *r* = 0.18, *p* = 0.32), PCC (controls *r* = 0.31, *p* = 0.06, migraineurs *r* = 0.19, *p* = 0.25), S1 (controls *r* = –0.001, *p* = 1.00; migraineurs *r* = 0.05, *p* = 0.76), dp insula (controls *r* = 0.19, *p* = 0.25; migraineurs *r* = 0.16, *p* = 0.34), hypothalamus (controls *r* = 0.20, *p* = 0.24; migraineurs *r* = 0.07, *p* = 0.68), putamen (controls *r* = 0.11, *p* = 0.53; migraineurs *r* = 0.01, *p* = 0.96) and V1 (controls *r* = 0.32, *p* = 0.06; migraineurs *r* = 0.08, *p* = 0.61). Extraction of gray matter volume from significant MD clusters revealed no overall group differences (Figures 3, 5; Table 4).

**Table 4:** Gray matter probability × volume and *p-*values for significant brainstem and wholebrain MD derived clusters in migraineurs and controls (corrected for multiple comparisons).

	***SpV***	***dlPons***	***PAG***	***PCC***	***S1***	***dp insula***	***hypothalamus***	***putamen***	***V1***
**Gray matter probability** **× volume [mean (±SEM)]**									
controls	0.20 ± 0.01	0.16 ± 0.01	0.38 ± 0.01	0.50 ± 0.01	0.15 ± 0.01	0.59 ± 0.01	0.31 ± 0.01	0.52 ± 0.01	0.61 ± 0.02
migraineurs	0.20 ± 0.01	0.18 ± 0.01	0.35 ± 0.01	0.50 ± 0.01	0.15 ± 0.01	0.59 ± 0.01	0.31 ± 0.01	0.52 ± 0.01	0.62 ± 0.01
significance	*p =* 0.99	*p =* 0.08	*p =* 0.06	*p =* 1.00	*p =* 0.94	*p =* 0.77	*p =* 0.30	*p =* 0.90	*p =* 0.84

## Discussion

This study demonstrates that the anatomy of the trigeminal nerve as assessed by FA is associated with microstructural changes in a number of brainstem and higher cortical brain regions in both pain-free controls and in migraineurs. Specifically, significant increases in MD within brainstem orofacial pain processing regions including the SpV, dlPons and PAG were found, consistent with our earlier study (5). Furthermore, these anatomical changes evident in the brainstem were also demonstrated in a number of higher pain processing brain regions such as the PCC, S1, dp insula, hypothalamus, putamen and V1. Interestingly, in almost all significant brain areas, both the control and migraineur groups displayed significant correlations between nerve and brain diffusivity suggesting that these relationships may not be influenced by the presence of migraine. However, there were two important exceptions: for both the PAG and V1 regions, FA of the trigeminal nerve was significantly correlated with diffusion in controls but not in migraineurs. This suggests that in these regions, migraine disrupts the normal relationship and that this may contribute to the underlying pathophysiology. Contrastingly, no changes in gray matter volume were demonstrated in brainstem and wholebrain areas which may indicate that alterations in the nerve represent more subtle microstructural changes that are not evidenced by overall gray matter volume within the brain of migraineurs during the interictal period.

Histological studies on the zygomaticotemporal branch of the trigeminal nerve have revealed altered structure in episodic migraineurs, including discontinuous and non-uniform proportions of myelin sheaths with disrupted axon targets (4). These nerve changes are consistent with a reduction of FA in the trigeminal nerve root entry zone in migraineurs that we demonstrated in a previous investigation (3). It is well known that the nerves innervating cerebral vasculature and dura enter the brain via the trigeminal nerve and terminate within the region that receives direct nociceptor afferents from the orofacial region, the SpV. The SpV projects directly to multiple brainstem sites involved in the processing of noxious information including the dlPons, more specifically the region of the parabrachial nucleus, and the PAG (18–20). We extend our previous trigeminal nerve results to show that in a number of brain regions, trigeminal nerve structure is correlated to structure within multiple brain sites in both controls and migraineurs.

As noted above, we have previously demonstrated overall MD increases in episodic migraineurs in SpV, dlPons and PAG (5), a finding that we confirm for the SpV and PAG in this study. In addition, we have found that when assessing all participants together, the anatomy of these brainstem regions display linear relationships with trigeminal nerve anatomy. Furthermore, whilst diffusivity of the SpV and dlPons were inversely correlated to nerve FA in both the control and migraine groups separately, for the PAG, there was a significant correlation only in the control group, i.e., in migraineurs no significant relationship was present. It is well known that the PAG modulates incoming noxious inputs at the level of SpV via projections to the rostral ventromedial medulla (21, 22). Indeed, experimental animal studies have shown that PAG stimulation inhibits SpV activity evoked by superior sagittal sinus stimulation (23). Furthermore, we have reported that acute orofacial noxious stimuli are associated with altered PAG activation and PAG- rostral ventromedial medulla signal coupling in migraineurs during the interictal phase (10). We speculate that our finding that MD in the PAG is elevated in migraineurs and that unlike controls, its diffusivity is decoupled to trigeminal nerve anatomy, is consistent with the idea that altered PAG function may reduce an individual's ability to dampen incoming noxious trigeminal information, which would increase the propensity for migraine attacks (23, 24).

Whilst the nature of the underlying cellular changes associated with increased MD are unclear, MD changes can be associated with multiple processes including inflammation, myelination, cell numbers and morphology (25, 26). We have previously shown that PAG diffusivity changes across the migraine cycle, increasing relative to controls during the interictal phase and decreasing to control levels immediately prior to a migraine attack (9). This dynamic response suggests that MD changes and relationships with the nerve in migraineurs may change relatively rapidly and may result from dynamic processes such as gliosis (27). There are several reports that suggest migraine is associated with reduced analgesic ability (28, 29), although some studies report no change (30, 31). This inconsistency may result from changes in analgesic ability across the migraine cycle which may be reflected in changes in the overall MD levels in the PAG and its relationship with trigeminal nerve anatomy.

In addition to altered analgesic ability, it has been proposed that in migraineurs, the internal brain state of dysregulation may result in an increased allostatic load. Allostatic load is the biological consequence that manifests as alterations in structure and/or function as a result of exposure to repeated stressful conditions (6). Whilst one could hypothesize that changes in higher brain regions correlated with altered trigeminal nerve anatomy in migraineurs may result from such a mechanism, the fact that in all higher brain regions apart from V1, both control and migraineurs displayed MD values that were correlated with nerve FA, suggests that these relationships are not tightly coupled to the presence of migraine itself. Instead, they are likely a basic property of the relationship between subtle variations in trigeminal nerve anatomy, possibly the make-up of fiber types, i.e., myelination, and subtle variations in the anatomy of brain regions that receive trigeminal information. This idea is supported by the fact that none of the higher brain regions that were correlated with nerve FA, for example, S1, dp insula, hypothalamus and putamen displayed differences between controls and migraineurs in overall mean MD. The circuitry between the trigeminal nerve and higher cortical areas has been demonstrated in experimental animal studies whereby orofacial nociceptive afferents synapse in the SpV and second order neurons then ascend to multiple brainstem and hypothalamic sites as well as via the the trigeminothalamic tract to the thalamus (18). From here, projections ascend to the S1 and other cortical areas such as the cingulate and insular cortices (32, 33).

The one cortical exception was the area of V1 which showed significantly increased overall diffusivity in migraineurs compared with controls and also showed significant linear relationships with nerve FA in the controls only, i.e., this relationship was not significant in migraineurs. Alterations in sensory processing regions such as V1 has recently been demonstrated in the interictal phase in episodic migraineurs (34). Indeed, we recently reported altered resting activity patterns in V1 in episodic migraineurs and also increased resting state functional connectivity between V1 and other sensory processing cortical regions including S1, primary auditory and insular cortices (34). It is posible that the disrupted relationship between trigeminal nerve FA and V1 diffusivity is associated with altered functional processing, even between V1 and other cortical areas, that ultimately precipitate photophobia and visual auras. Alternatively, although the majority of the participants in the current study had migraine without aura, sensitivity to light during a migraine attack was reported in more than half of the migraine participants. We might expect that underlying anatomical alterations in V1 of migraineurs might contribute to the abnormal sensitivity to light that appear to be present in the symptom-free period.

There are a number of limitations to this study that need to be acknowledged. Given that the spatial resolution of human brain imaging techniques, particularly DTI, is relatively low, it is difficult to determine the precise location of each significant cluster with respect to small brainstem nuclei and cortices. However, the location of each significant cluster was identified using brain and brainstem atlases in conjunction with existing human and preclinical literature. Future longitudinal studies exploring the structural changes within the brainstem and cortical regions over the different phases of migraine are warranted to confirm the cross-sectional findings in this study which would provide a greater understanding about the nature of the structural changes and if such changes are evident across the entire migraine cycle. Furthermore, given the cross-sectional design of this study, it is difficult to delineate the precise biological mechanisms underlying the correlations that we have found between the nerve and brain areas. Corroborating our findings with other literature including experimental animal studies and histological studies, would provide an improved understanding and interpretation of these mechanisms.

Overall, our findings suggest there is an anatomical relationship between the trigeminal nerve and its recipient regions in the brainstem and higher brain regions. These relationships appear to be present equally in controls and migraineurs in most regions, with the notable exception of the PAG as well as the V1. Understanding these underlying anatomical changes may inform the underlying pathophysiology of migraine, which could aid in the development of targeted therapeutics.

## Data availability statement

The datasets presented in this article are not readily available because data was collected under an ethics approval that did not allow for data sharing. Requests to access the datasets should be directed to luke.henderson@sydney.edu.au.

## Ethics statement

The studies involving human participants were reviewed and approved by Local Institutional Human Research Ethics Committee. The patients/participants provided their written informed consent to participate in this study.

## Author contributions

TM performed the data analysis and wrote the original draft and final version. NM, LH, and VM were involved in study design and data collection. PM was involved in data analysis. LH edited all versions of the manuscript following the initial draft. All authors contributed to the article and approved the submitted version.

## Funding

This work was supported by grants (1032072 and 1059182) awarded by the National Health and Medical Research Council of Australia.

## Conflict of interest

The authors declare that the research was conducted in the absence of any commercial or financial relationships that could be construed as a potential conflict of interest.

## Publisher's note

All claims expressed in this article are solely those of the authors and do not necessarily represent those of their affiliated organizations, or those of the publisher, the editors and the reviewers. Any product that may be evaluated in this article, or claim that may be made by its manufacturer, is not guaranteed or endorsed by the publisher.
