# Supplementary figures and images for: Alterations in brain structure associated with trigeminal nerve anatomy in episodic migraine
Source: Front Pain Res (Lausanne). 2022 Jul 18;3:951581. doi: 10.3389/fpain.2022.951581 (PMC9341524; doi:10.3389/fpain.2022.951581)

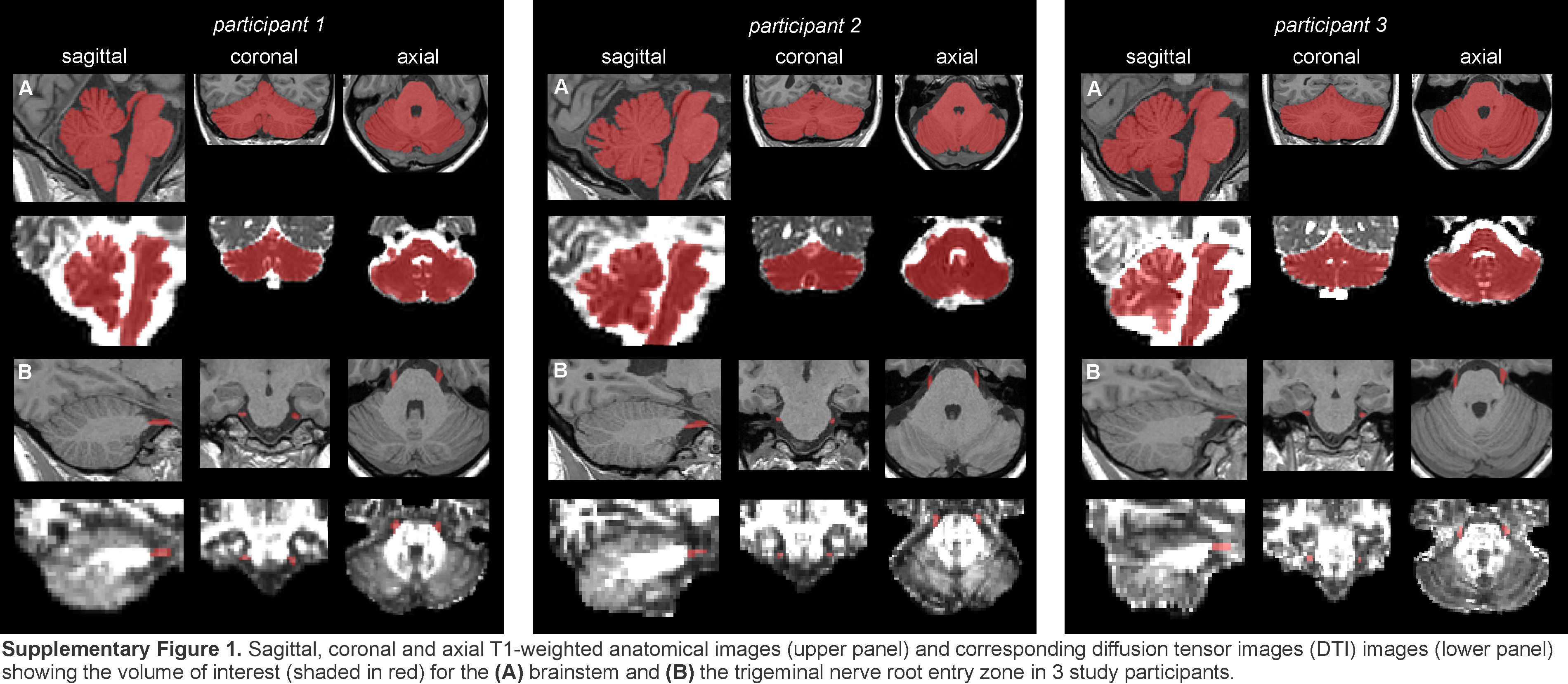

Supplement: Supplementary file 1 [file Image_1.JPEG]
